# A cross-sectional study in critically ill patients affected by COVID-19 in an intensive care unit: would *Acinetobacter baumannii* resistant to carbapenems be the biggest villain?

**DOI:** 10.1099/acmi.0.000542.v3

**Published:** 2023-10-18

**Authors:** Sayuri Rocha Yamashita, Vera Lucia Milani Martins, Elves Anderson Pires Maciel, Francine Johansson Azeredo

**Affiliations:** ^1^​ Graduate Program in Pharmaceutical Assistance of Federal University of Bahia. R. Barao de Jeremoabo, 147, 40170-115, Salvador, BA, Lucia, Brazil; ^2^​ Hospital Santa Izabel. Praca Conselheiro Almeida Couto, 500, Salvador, BA, 40050-410, Brazil; ^3^​ Federal Institute of Education, Science, and Technology of Rio Grande do Sul. R. Cel. Vicente, 281, Porto Alegre, RS, 90030-041, Brazil; ^4^​ Center for Pharmacometrics and Systems Pharmacology, Department of Pharmaceutics, College of Pharmacy, University of Florida, Orlando, FL, 32827, USA

**Keywords:** hospitalized patients, ICU, resistance, SARS-COV-2

## Abstract

Aim: to describe cases of infection of *Acinetobacter baumannii (A. baumannii*) in critically ill patients affected by COVID-19, admitted to an intensive care unit (ICU), using dexamethasone. Methodology: cross-sectional study conducted on patients admitted to the intensive care unit COVID-19 survey among hospitalized patients from November 2020 to March 2021. Setting: large hospital, reference for caring for patients with COVID-19 in Bahia (Brazil). Patients: a convenience sample of 22 patients admitted to the COVID ICU signed the consent form agreeing to participate in the study. Three patients were excluded for having decided to participate without signing the form. Results: of the 22 patients listed, 45 % (10) had blood infection or mechanical ventilation-associated pneumonia by *A. baumannii* in blood cultures and/or tracheal aspirate secretion. We observed that there is a moderate correlation between the length of stay and infection by *A. baumannii* (Spearman’s ρ; 0.592; *p*-value<0.005) and a strong correlation between the number of days on mechanical ventilation and infection by these bacteria (Spearman’s ρ; 0.740; *p*-value<0.001). This percentage is higher than the value of 0.62 % of infection by *A. baumannii* in this ICU in the same period of the year before COVID-19 (*p*-value<0.0001). Conclusions: hospitals that receive patients with COVID-19 may be vulnerable to outbreaks of multi-drug resistant organisms, such as *A. baumannii *. It is worth reflecting on the care and operational practices in handling these patients, especially in isolation and restriction measures for those from other nosocomial areas.

## Data Summary

The authors confirm that all supporting data, code, and protocols have been provided within the article.

## Introduction

COVID-19 is a severe acute respiratory syndrome caused by SARS-CoV-2, first identified in Wuhan, China, in December 2019 and declared a pandemic on 11 March 2020 [1]. The disease can manifest in patients with varying severity, ranging from asymptomatic cases to those requiring hospitalization due to respiratory impairment. Severe cases often necessitate intensive care with mechanical ventilation support [2].

As of 9 October 2022, the cumulative number of confirmed COVID-19 cases worldwide has exceeded 618 million, resulting in approximately 6.5 million deaths. Despite the significant impact, there is still no proven pharmacotherapy to cure the virus. Several therapeutic approaches have been proposed to address the inflammatory lesions associated with viral pneumonia caused by COVID-19, and one such option is the use of glucocorticoids. Notably, Horby *et al*. [3] highlighted the efficacy of the corticosteroid dexamethasone in a study involving 2104 hospitalized patients who received a daily dose of 6 milligrams of dexamethasone for ten days. A comparative analysis with 4321 patients who received standard care demonstrated a 29.3 % mortality rate in the dexamethasone group compared to 41.4 % in the usual care group after 28 days. The study revealed that dexamethasone was responsible for a significant reduction in mortality by 29 % in patients on mechanical ventilation. However, the benefits were less pronounced for patients who only received oxygen support, with a mortality reduction of 23.3 % in the dexamethasone group compared to 26.2 % in the standard care group. Additionally, caution is advised regarding the early use of dexamethasone in patients not requiring oxygen at the start of treatment, as it may increase mortality risk [3].

The pandemic has brought several challenges, including shortages of healthcare professionals, personal protective equipment, and hospital supplies. Disruptions in conventional infection prevention and control practices have highlighted the potential risk of spreading multiresistant organisms [4]. Among the complications associated with COVID-19, patients requiring mechanical ventilation are at risk of developing ventilation-associated pneumonia (VAP), mainly from multiresistant bacteria like *Acinetobacter baumannii* [5]. Patients with severe COVID-19 often possess the main risk factors observed for VAP caused by *A. baumannii *, such as hypertension, chronic obstructive pulmonary disease, chronic renal failure, prolonged ICU stays, organ failure, and low blood oxygenation [5, 6].

Given the current challenges and scenario, this study aims to describe cases of *A. baumannii* co-infection in critically ill patients affected by COVID-19 who were admitted to an intensive care unit and received dexamethasone as part of their treatment.

## Methods

A cross-sectional study was conducted on patients admitted to the COVID-19 intensive care unit of a designated COVID reference hospital in Salvador, Bahia. The Research Ethics Committee of the hospital approved the study, and patients or their guardians provided informed consent via email, in person, or through Google Forms, allowing the utilization of data from medical records and blood samples. Blood samples were collected from these patients in EDTA tubes after administering dexamethasone to assess their laboratory profile.

Inclusion criteria for the study were adult patients over 18 years old, confirmed to have the viral infection SARS-CoV-2, who agreed to participate by signing the consent form and were using dexamethasone, as it was the only pharmacotherapy with proven efficacy at the time of analysis. This research is part of a broader study that aims to quantify the serum levels of this drug. Patients under 18 years old and those who disagreed with the investigation were excluded. Patients’ demographic data were collected at the time of admission from electronic medical records.

Quantitative variables were described using the mean to indicate central tendency and the standard deviation to describe variability. Qualitative variables were defined using counts and percentages. Z-test was used to evaluate rates in comparison to reference values.

Correlation analyses were conducted using Pearson’s correlation for quantitative variables and Spearman’s ρ for evaluating ordinal variables. Infections caused by *A. baumannii* in the ICU were compared with historical data from the same period before the COVID-19 pandemic. Also, the number of days on mechanical ventilation was compared with the incidence of *A. baumannii* infections.

Difference tests between distributions were performed using Kruskal-Wallis or Mann-Whitney U test when normality assumptions could not be met. Association tests were conducted using the chi-square test. Pearson’s correlation analysis was used to observe correlations among quantitative variables. Relative Risk (RR) analyses were applied to identify an increased risk for mortality and infection with *A. baumannii *. Statistical significance was set at α=0.05, and the test’s estimated significance was calculated using a two-tailed *p*-value<0.05.

### Results

Of the 22 patients listed for the study, 45 % (10) were infected by *A. baumannii* in blood cultures and/or tracheal aspirate secretion. Notably, this percentage is significantly increased compared to the history of approximately 0.62 % of infections by *A. baumannii* in the ICU in the same period in the year before COVID-19 (*p*-value<0.0001). It was observed that there is a moderate correlation between the length of stay in the ICU and infection by *A. baumannii* (Spearman’s ρ; 0.592; *p*-value<0.005) and a strong correlation between the number of days on mechanical ventilation and infection by *A. baumannii* (Spearman’s ρ; 0.740; *p*-value<0.001).

As for the profile of patients infected by *A. baumannii *, 60 % are male and 40 % female, with a mean age of 66.4 years and a standard deviation of 11.59 years. These patients, on average, were hospitalized for 19.6 days in the ICU-COVID until the outcome, with a standard deviation of 8.62 days. At the same time, the mean time from admission to this unit and installation of mechanical ventilation until the moment of collection of the culture were 10.6 and 10 days, with standard deviations of 6.16 and 5.31 days, respectively. In addition to *A. baumannii *, other pathogens coinfected several patients (Table 1).

**Table 1.** Patients’ demographic and laboratory

Patient	Sex	Age range (years)	ICU time (days)	Creatinine (according to reference)	Dialysis (Y/N)	Deceased (Y/N)	CMV or CSV	CVM time to culture collection (days)	Culture type	Microorganism
1	Female	65–70	5	High	N	Y	CMV	0	Hemoculture	*Candida tropicalis*
2	Female	70–75	16	Normal	N	N	CMV	5	Hemoculture	* Enterococcus faecalis* and Coagulase-negative *Staphylococci*
3	Female	65–70	11	Normal	N	N	CMV	0	Hemoculture	Coagulase-negative *Staphylococci*
4	Male	55–60	10	Normal	N	Y	CMV	0	Not collected	
5	Female	60–65	8	Normal	N	N	CSV	0	Hemoculture, urine culture, and tracheal aspirate	None
6	Male	30–35	6	Normal	N	N	CSV	0	Hemoculture, urine culture, and tracheal aspirate	None
7	Male	45–50	34	Normal	N	N	CMV	10	Hemoculture and urine culture	Multidrug-resistant *Acinetobacter baumannii* and multidrug-resistant *Klebsiella pneumoniae *
8	Male	25–30	9	Normal	N	N	CMV	7	Tracheal aspirate	* Pseudomonas aeruginosa *
9	Female	35–40	8	High	N	Y	CMV	7	Hemoculture	Coagulase-negative *Staphylococci*
10	Male	85–90	14	Normal	N	N	CMV	6	Tracheal aspirate	Multidrug-resistant *Acinetobacter baumannii *
11	Male	85–90	9	High	N	Y	CMV	0	Hemoculture tracheal, urine culture, and aspirate	None
12	Male	60–65	23	Normal	N	N	CMV	14	Hemoculture and tracheal aspirate	Multidrug-resistant *Acinetobacter baumannii* and *Pseudomonas aeruginosa *
13	Male	70–75	12	Normal	N	N	CMV	6	Hemoculture and tracheal aspirate	Multidrug-resistant *Acinetobacter baumannii *
14	Male	50–55	7	High	Y	Y	CMV	6	Hemoculture and tracheal aspirate	Multidrug-resistant *Acinetobacter baumannii *
15	Male	40–45	8	Normal	N	N	CSV	0	Hemoculture, Urine culture, and tracheal aspirate	None
16	Male	70–75	11	High	Y	Y	CMV	7	Tracheal aspirate	Multidrug-resistant *Acinetobacter baumannii *
17	Female	60–65	28	High	Y	Y	CMV	8	Hemoculture and tracheal aspirate	Multidrug-resistant *Acinetobacter baumannii* and *Pseudomonas aeruginosa *
18	Female	80–85	19	High	Y	Y	CMV	8	Urine culture and tracheal aspirate	Multidrug-resistant *Acinetobacter baumannii* and *Enterococcus faecalis *
19	Female	65–70	22	Normal	N	N	CMV	6	Tracheal aspirate	Multidrug-resistant *Klebsiella pneumoniae *
20	Female	80–85	9	Normal	N	Y	CMV	0	Urine culture	Multidrug-resistant *Klebsiella pneumoniae *
21	Female	60–65	27	Normal	N	N	CMV	23	Tracheal aspirate	Multidrug-resistant *Acinetobacter baumannii *
22	Female	55–60	21	High	N	N	CMV	12	Hemoculture	Multidrug-resistant *Acinetobacter baumannii,* * Pseudomonas aeruginosa* and *Enterococcus faecalis *

CMV, Controlled mechanical ventilation; CSV, Continuous spontaneous ventilation.

In patients infected by *A. baumannii *, regarding the description of pre-existing comorbidities, 80 % had arterial hypertension or diabetes. In addition, 30 % had previous kidney disease, and 20 % developed the condition during hospitalization. In our group of patients, the ones with renal dysfunction who progressed to hemodialysis died (40 %). Still, it was impossible to identify a Significant Relative Risk (RR) for this factor in this sample. Considering creatinine as a marker of renal dysfunction, a relationship with the outcome was observed in patients positive for infection by *A. baumannii *. From this perspective, it was possible to identify a strong correlation between creatinine values and the outcome (Spearman’s ρ; 0.782; *p*-value<0.008) while considering the same analysis including non-colonized patients, a moderate correlation was observed between these values (Spearman’s ρ; 0.561; *p*-value<0.007). On the other hand, it was not possible to identify a significant RR between increased creatinine and infection by *A. baumannii *. Table 1 presents more details about the patients included in the study.

### Discussion

Regarding the critical culture findings, carbapenem-resistant *A. baumannii* causes healthcare-associated infections that are difficult to contain and are often associated with infection prevention and control violations, particularly among critically ill patients [7]. From February to July 2020, a New Jersey Hospital and the Department of Health investigated and identified 34 patients with multidrug-resistant or previous colonization-acquired *A. baumannii *. From this sample, 28 (82 %) patients had infection or colonization during increased installation in COVID-19 cases (from March to June 2020), and 17 (50 %) confirmed infection with SARS-CoV-2, the virus that causes COVID-19. The mean age of patients with carbapenem-resistant *A. baumannii* infection was 55 years. Twenty-five (74 %) patients were intubated and mechanically ventilated at the time of sample collection. Twenty-three (68 %) patients with CRAB infection had been discharged, and 10 (29 %) had died [4]. Patients with mechanical ventilation have a higher risk of acquiring bacterial pneumonia in the ICU, so the associated cases of COVID-19 were considerably more severe, possibly worsening the clinical scenario and requiring special attention from health professionals [5, 8, 9].

A case series included 5700 patients hospitalized with COVID-19 in New York, whose average age was 63 years, and 39.7 % were women, who had hypertension (56.6 %), obesity (41.7 %) and diabetes (33.8 %) as the most common comorbidities. Among patients who were discharged or died (14.2 %) were treated in the intensive care unit, 12.2 % received invasive mechanical ventilation, 3.2 % were treated with renal replacement therapy, and 21 % died, consistent with reported changes by Huang *et al*. [2] who include underlying structural heart disease, cardiac injury, kidney, and liver dysfunction [7].

According to this study, in patients infected by *A. baumannii *, 80 % of the patients had arterial hypertension or diabetes. In addition, 30 % had previous kidney disease, and 20 % developed the condition during hospitalization. These results were consistent with case series and literature records of older adults and people with pre-existing hypertension and/or diabetes in the USA [10]. The literature indicates that hemodialysis patients have an increased risk of death [10]. In our group of patients, the ones with renal dysfunction who progressed to hemodialysis died (40 %). It is still unclear whether COVID-19 is directly linked to kidney damage or results from complications arising from a viral infection outside the kidney. However, like most kidney diseases, the mechanisms are probably multifactorial, and data so far point to contributions from a direct viral infection, inflammatory syndrome-mediated injury, and haemodynamic instability. They are associated with increased in-hospital mortality [11].

Another study conducted in Qom, Iran, focused on 19 critically ill patients admitted to two hospitals for coronavirus treatment in their intensive care units (ICUs). Among these patients, 11 (58 %) were male, and eight (42 %) were female, with an average age of approximately 67. The mean length of stay in the ICU was about 15 days. After the study, 18 cases (95 %) resulted in fatalities, while only one case (5 %) survived. All patients tested positive for bacterial infections, including 17 strains of *A. baumannii* (90 %) and two strains of *Staphylococcus aureus* (10 %). There was no variation in bacterial species detected at different sampling points. All *A. baumannii* strains were found to be resistant to the antibiotics under evaluation, and no metallo-beta-lactamase-producing strain of *A. baumannii* was found [12].

It is important to note that carbapenem-resistant *A. baumannii* can colonize individuals for extended periods, indicating that they may not have acquired the infection solely during their stay in the ICU under study. Our research involved blood culture and tracheal aspirate collections from COVID-19 ICU patients for over 6 days. This unit received patients from various institutions and emergency care units during the pandemic, suggesting a possibility of previous infections. In a study conducted in China, five out of 36 ICU-admitted patients (13.9 %) were diagnosed with severe acute respiratory syndrome due to coronavirus and secondary bacterial infections [13]. Similarly, in a report from a UK secondary care setting, among 836 identified SARS-CoV-2 patients, 27 cases (3.2 %) had confirmed bacterial isolates detected early (within 0 to 5 days after admission), and this number increased to 51 cases (6.1 %) during hospitalization [14].

One possible reason for the increased infection rate could be the virus’s and bacteria’s concurrent infection, facilitating their fixation and colonization in the respiratory tract. This can lead to worse clinical outcomes and increased mortality following viral respiratory infections, including in patients with COVID-19 who develop ventilator-associated pneumonia (VAP). According to the study, impaired antibacterial immune defence can be detected weeks before the onset of secondary infection. These findings may also have significant implications for patients treated with corticosteroids, as they may further suppress the critical pathways required for the host response to secondary bacterial infection [15].

Additionally, various factors such as the type of unit where the patient is hospitalized, equipment used, admission or discharge criteria, high workload or shortage of healthcare professionals, and scarcity of materials and medicines can also impact the quality of care and the rate of nosocomial infections acquired in the ICU, particularly during pandemics [4, 10].

These results underscore the concern of bacterial infections in patients, with *A. baumannii* showing resistance to extended-spectrum antibiotics typically used to treat life-threatening bacterial diseases, especially in critically ill patients who move between different inpatient units. Therapeutic management of these patients involves monotherapy with polymyxin or combined therapy, which has proven to be more effective in treating patients with COVID-19 and co-infections [16]. According to our antibiogram results, all isolated *A. baumannii* strains were resistant to carbapenems and polymyxin, with 30 % showing resistance to gentamicin and only 20 % to amikacin.

When comparing significant morbidity and mortality rates with other non-COVID-19 studies, it is suggested that mortality increases in COVID-19 patients and can be attributed to bacterial coinfections, including in those patients without underlying diseases, such as the study carried out in Shiraz and Mysuru whose death rate of non-COVID patients were respectively 10.9 and 17.8 % [12, 17, 18]. Toufen *et al*. [19] report that the mortality rate in an ICU was 28.8 %, while patients with infection had a mortality rate of 34.7 %. *A. baumannii* infection may be associated with considerable mortality, varying from 8–35 %, and a Brazilian review summarized that the prevalence of *A. baumannii* infection was 32.8 % in Brazil in this pandemic period [20]. Another retrospective multicenter study in Shanghai indicated overall mortality of 32.47 % associated with VAP, whose primary pathogens were *A. baumannii* (33.96 %), *Klebsiella pneumoniae* (23.58 %), *Pseudomonas aeruginosa* (19.81%), and *Staphylococcus aureus* (7.08 %) [2].

A study in China reported that 13.9 % of critically ill patients with coronavirus disease 2019 (COVID-19) in a COVID ICU developed secondary bacterial infections. All five out of 36 patients were admitted to the intensive care unit and received mechanical ventilation before the onset of bacterial infections. Clinical manifestations, such as fever or hypothermia, purulent respiratory secretions, new or progressive pulmonary infiltrates on chest radiography, and elevated peripheral leukocytes or C-reactive protein or procalcitonin levels, characterized these infections. Like our present study, all five patients were aged ≥55 years old, and 80 % were male. The mean time from ICU admission and mechanical ventilation to the onset of bacterial co-infection was 11 days and 8.6 days, respectively, compared to 10.6 days and 10 days, respectively [13].

It is essential to acknowledge some limitations in drawing conclusions from this study, mainly the relatively small number of patients. We did not perform whole genome sequencing to determine the relatedness of isolates. Additionally, the severe pandemic conditions and study design made having a control group without COVID-19 in the ICU impossible. Consequently, we cannot definitively attribute the percentages of deaths in patients solely to nosocomial bacterial infections. However, this study prompts reflection on the care and operational practices when handling these patients, especially during a pandemic, and the use of empirical antibiotics at the onset of COVID-19 symptoms. Another critical issue arising from the COVID-19 pandemic is the isolation and restriction measures for patients from other healthcare facilities.

Hospitals that receive patients with COVID-19 may be vulnerable to outbreaks of multi-drug-resistant organisms. Therefore, it is essential to limit the risk of infections and the spread of these resistant strains by implementing precise control measures and increasing attention to secondary infections caused by resistant bacteria, which can elevate the mortality rate in critically ill COVID-19 patients.
